# Apoptosis rate and transcriptional response of pancreatic islets exposed to the PPAR gamma agonist Pioglitazone

**DOI:** 10.1186/1758-5996-5-1

**Published:** 2013-01-08

**Authors:** Rodrigo N Lamounier, Cassio N Coimbra, Peter White, Flavia L Costal, Leonardo S Oliveira, Daniel Giannella-Neto, Klaus H Kaestner, Maria Lúcia Corrêa-Giannella

**Affiliations:** 1 Laboratory for Cellular and Molecular Endocrinology LIM-25, University of Sao Paulo Medical School, Av. Dr. Arnaldo 455 #4305, 01246-903, São Paulo, Brazil; 2 Department of Genetics and Institute for Diabetes, Obesity and Metabolism, University of Pennsylvania School of Medicine, 752B CRB 415 Curie Blvd., 19104, Philadelphia, Pennsylvania, USA; 3 Laboratory Medicine, University of Santo Amaro, Sao Paulo, Brazil; 4 Postgraduate Program in Medicine, Universidade Nove de Julho - Uninove, Sao Paulo, Brazil

**Keywords:** PPARγ, Pioglitazone, Islets, Gene expression, Apoptosis

## Abstract

To explore the molecular pathways underlying thiazolidinediones effects on pancreatic islets in conditions mimicking normo- and hyperglycemia, apoptosis rate and transcriptional response to Pioglitazone at both physiological and supraphysiological glucose concentrations were evaluated. Adult rat islets were cultured at physiological (5.6 mM) and supraphysiological (23 mM) glucose concentrations in presence of 10 μM Pioglitazone or vehicle. RNA expression profiling was evaluated with the PancChip 13k cDNA microarray after 24-h, and expression results for some selected genes were validated by qRT-PCR. The effects of Pioglitazone were investigated regarding apoptosis rate after 24-, 48- and 72-h. At 5.6 mM glucose, 101 genes were modulated by Pioglitazone, while 1,235 genes were affected at 23 mM glucose. Gene networks related to lipid metabolism were identified as altered by Pioglitazone at both glucose concentrations. At 23 mM glucose, cell cycle and cell death pathways were significantly regulated as well. At 5.6 mM glucose, Pioglitazone elicited a transient reduction in islets apoptosis rate while at 23 mM, *Bcl2* expression was reduced and apoptosis rate was increased by Pioglitazone. Our data demonstrate that the effect of Pioglitazone on gene expression profile and apoptosis rate depends on the glucose concentration. The modulation of genes related to cell death and the increased apoptosis rate observed at supraphysiological glucose concentration raise concerns about Pioglitazone’s direct effects in conditions of hyperglycemia and reinforce the necessity of additional studies designed to evaluate TZDs effects on the preservation of β-cell function in situations where glucotoxicity might be more relevant than lipotoxicity.

## Introduction

Type 2 diabetes is characterized by impaired insulin secretion and reduced peripheral insulin sensitivity. Loss of β-cell function causes not only the progression from the prediabetic state to overt disease, but also hastens the deterioration of metabolic control in people with type 2 diabetes [1,2]. Thiazolidinediones (TZDs), a family of peroxisome proliferator-activated receptor γ (PPARγ) ligands, act by improving peripheral insulin sensitivity [3]. Clinical studies have shown that treatment with TZDs maintains β-cell function and prevents high risk patients from progressing to diabetes [4-6]. Studies in rodents demonstrated that these drugs reduce β-cell impairment and decrease islet fatty acid accumulation [7,8]. It is known that activation of PPARγ *in vivo* induces several peripheral adaptations that result in improved insulin sensitivity and reduced insulin demand, however, if there are direct effects such as reduced lipotoxicity [9,10], protection from oxidative stress and from apoptosis [11] remains to be further investigated.

Apoptosis constitutes the main form of β-cell death [12] and gluco- and/or lipotoxicity are two of the major mechanisms for islets dysfunction and apoptosis in pancreatic cells in type 2 diabetes [13]. While TZDs have been reported to have direct beneficial effects on β-cells by preventing these toxicities [14,15], by promoting antioxidative effects [16] and by preventing β-cell dysfunction under conditions of concomitant hyperglycemic and cytokine stress [17], this notion has been contested by others [18,19]. To further explore the molecular pathways underlying TZDs direct effects on pancreatic islets in conditions mimicking normo- and hyperglycemia, we have determined transcriptional response and apoptosis rate of rat islets to Pioglitazone, currently the only TZD in clinical use, at both physiological and supraphysiological glucose concentrations.

## Methods

### Islets isolation and culture

Islets were isolated from male Wistar rats (2 months of age, 220-260 g) after perfusion of the pancreatic duct, collagenase (Type V) digestion and purification on Ficoll gradients [20] and cultured in RPMI-1640 media with 10% FCS, 5.6 mM glucose, 100 IU/ml penicillin and 100 μg/ml streptomycin. All reagents were obtained from Sigma-Aldrich Chemical (St. Louis, MO, USA). All animal procedures were in accordance with NIH’s ‘Principles of laboratory animal care’, and approved by the local ethics committee.

### Expression profiling

After 24 h of isolation, approximately 250 islets were cultured with 10 μM Pioglitazone (Takeda Pharmaceuticals, Japan) or DMSO (vehicle) for 24 h in either physiological (5.6 mM) or supraphysiological (23 mM) glucose concentrations. Both control and treated culture media contained 0.1% DMSO as a final concentration.

**Figure 1:**
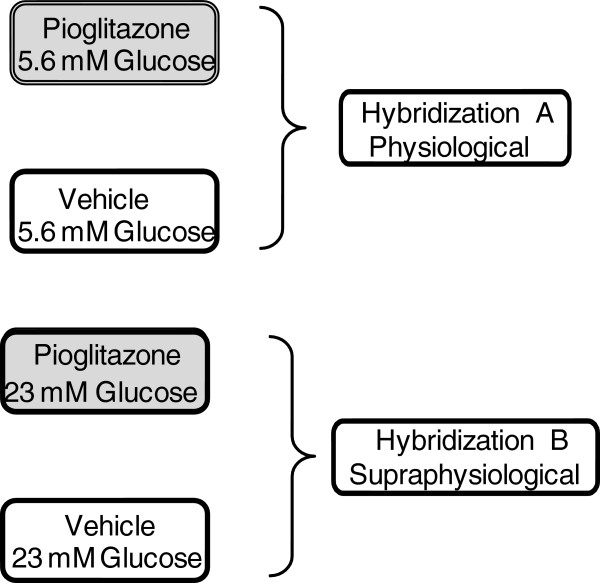
**Microarray hybridization setup.** Two experiments were performed in parallel, at 5.6 mM (physiological) and 23 mM (supraphysiological) glucose concentrations.

Two array experiments were performed in parallel to analyze the effect of Pioglitazone at both glucose concentrations (Figure 1). A direct comparison design was used, such that hybridizations were set up as Test (Cy5) vs. Control (Cy3). Five biological replicates were used for each condition.

All RNA samples were analyzed using an Agilent Bioanalyzer Lab-on-a-Chip Nano 6000 chip to determine the integrity and concentration of the samples. Only samples with a RIN factor > 6.0 were used. Five μg of total RNA was indirectly labeled using amino-allyl dUTP and an anchored oligo (dT)_20_ to prime reverse transcription. Methods for fluorescent labeling and data acquiring were as described [21]. The high level of sequence similarity between mouse and rat genes makes the Mouse PancChip array suitable for use with rat tissue [22]. Statistical analysis was performed in “R” using both the LIMMA [23] and Statistical Analysis of Microarrays (SAM) package (http://www-stat.stanford.edu/~tibs/SAM/) [24]. A one-class unpaired analysis with a False-discovery rate (FDR) of 20% was employed. The list of significantly differentially expressed genes was filtered to remove genes with an absolute change ≤ 1.2 fold. Microarray data are available through the Minimum Information About a Microarray Experiment (MIAME, accession number GSE40119) [25]. Biologically relevant networks were drawn from the two lists of differentially expressed genes identified by SAM analysis. Ingenuity Pathways Analysis (http://www.Ingenuity.com) was employed to analyze networks affected by Pioglitazone as described [21].

### Quantitative real-time reverse transcription PCR (qRT-PCR)

Differential gene expression was confirmed using qRT-PCR, as previously described [21] on RNA prior to amplification and using specific primers designed for rat genes (Additional file 1: Table S1). *Gapdh, Actb*, *Hprt* and *Tbp* were tested for their potential use as housekeeping genes using the geNorm analysis package [26], and *Gapdh* was selected as the most stable. Relative levels of mRNA expression were calculated using the 2^ΔΔCT^ method [27]. Five independent experiments were performed in duplicate.

### Evaluation of apoptosis

To evaluate the chronic effect of Pioglitazone on apoptosis, islets were cultured in RPMI-1640 with 10% FCS and exposed or not (vehicle) for 24, 48 and 72 h to 10 μM Pioglitazone at 5.6 mM or 23 mM glucose. qRT-PCR was performed as described above to analyze the expression of the antiapoptotic gene *Bcl2*. The rate of apoptosis was evaluated by quantification of DNA fragmentation with the Cell Death Detection Enzyme-Linked Immunosorbent Assay Plus Kit (Roche Molecular Biochemistry, Germany). Aliquots of cytoplasmic lysates (50 islets/reaction) were analyzed and quantified using an Anthos' LUCY-3 luminometer (Anthos Labtec Instruments, Australia) at 405 nm. Comparison of the absorbance of treated with untreated samples determined the extent of DNA fragmentation. Absorbance values from the samples were corrected for the background values and the results were expressed as arbitrary units (AU). To confirm the increased rate of apoptosis in islets exposed to Pioglitazone and 23 mM glucose at 48 and 72 h, caspase-3 enzyme activity was measured using the ApoTarget Caspase-3 Protease Assay Kit (BioSource International Inc, USA). Aliquots of cytoplasmic lysates (1,000 islets/reaction) were analyzed and quantified using an Anthos' LUCY- luminometer at 405 nm. Results were corrected for protein content and expressed as AU. Three independent experiments were performed in triplicate.

To rule out a toxic effect of Pioglitazone, batches of 10 islets were cultured in RPMI-1640 with 10% FCS and exposed or not (vehicle) for 24 h to 10 μM Pioglitazone at 5.6 mM glucose and at 23 mM. The insulin secretory response was studied after islet incubation for 1 h in Kreb’ s buffer with 1.6 mM glucose for stabilization, followed by incubation for 1 h at 1.6 or 16.7 mM glucose. Incubation media were collected for insulin measurement by ELISA (Linco Research, St Louis, USA). Results were corrected for DNA content and stimulation indices were calculated by dividing insulin secretion at high glucose by basal insulin secretion. Three independent experiments were performed in duplicate.

### Statistical analysis

Statistical tests were performed using the JMP Version 6.0 statistical computer program (SAS Institute). The Student’s t test was used to confirm that the qRT-PCR results were significant and matched the direction of the fold change predicted by the array. Because assumptions for a parametric test were not valid (P < 0.05, Kolmogorov-Smirnov), all data concerning apoptosis rate and measurement of insulin content were evaluated by Wilcoxon-Mann-Whitney after quantile transformation (Q), i.e., quantile data transformation is used for the construction of the quantile-quantile (Q-Q) where the values in the sample of data, in order from smallest to largest, are denoted x(1), x(2), …, x(n). For i = 1, 2, ....., n, x(i) is plotted against F-1((i-0.5)/n). Data were expressed as median and semi-interquartile range. Statistical significance was fixed at probability levels of < 0.05.

## Results

### The transcriptional response of the islet to TZDs

The transcriptional response of pancreatic islets to Pioglitazone was determined at normal (5.6 mM) and high (23 mM) glucose concentrations using the PancChip microarray. Expression of 101 genes was modulated by Pioglitazone at 5.6 mM of glucose, with 49 upregulated by the drug and 52 downregulated. At 23 mM glucose 1,235 genes were affected, with 612 uregulated by the drug and 623 downregulated. The list of all genes modulated by Pioglitazone at 5.6 mM and the list of the 200 first genes down- and up-regulated by Pioglitazone at 23 mM are shown on Additional file 2: Tables S2 (5.6 mM) and Additional file 3: Table S3 (23 mM).

The microarray results were validated using qRT-PCR, which closely mirrored the results of the array analysis (Table 1). We also evaluated five genes previously suggested as PPARγ targets, *Gck*, *Slc2a2*, *Abca1*, *Lpl* and *Ucp2*, as well as the expression of *Pparg*, *Ppara*, *Ins1* and *Ins2*. None of these genes were Pioglitazone responsive in islets as determined by qRT-PCR (data not shown).

**Table 1:** Confirmation (by qRT-PCR) of gene expression changes induced by 24-hour exposure to Pioglitazone at 5.6 mM and 23 mM glucose concentrations

	**Genes**	**PanChip**		**qRT-PCR**	
		**FC**	**FDR**	**FC**	***p*-value**
5.6 mM	*Fabp4*	1.6	14.57	5.8	0.02
	*Insig1*	1.4	14.57	2.0	0.02
	*Scd1*	1.5	14.57	1.2	0.32
	*Scd2*	1.4	14.57	1.9	0.03
	*Sc4mol*	1.3	17.95	1.2	0.44
23 mM	*Actb*	1.4	6.5	1.6	0.17
	*Bad*	1.4	5.0	1.4	0.05
	*Bax*	1.3	4.0	1.5	0.02
	*Camk2b*	1.3	9.3	1.2	0.42
	*Fabp4*	1.4	4.0	5.0	0.02
	*Hmox1*	1.4	7.3	3.4	0.04
	*ltpr2*	1.3	4.0	1.5	0.04
	*Npy*	−1.3	2.3	−2.7	0.19
	*Prdx6*	1.4	11.4	1.3	0.34
	*Sod2*	1.4	6.7	4.5	0.02

Positive and negative FC (fold-change) represent, respectively, genes upregulated and downregulated by Pioglitazone. FDR: false-discovery rate.

**Figure 2:**
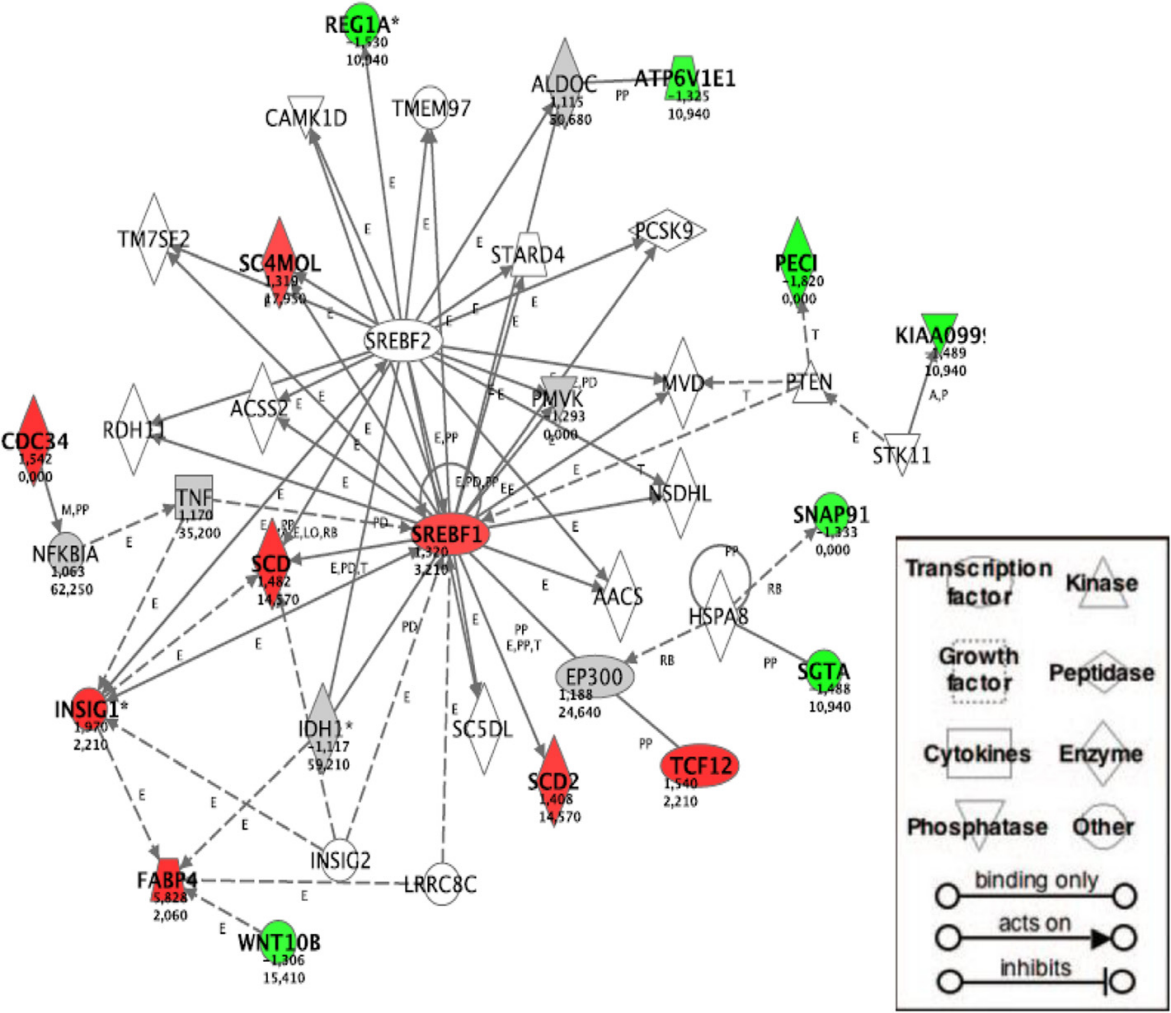
**Pathway analysis identifies a network of genes related to lipid metabolism in islets cultured at 5.6 mM glucose concentration.** The network is displayed graphically as nodes (genes/gene products) and edges (the biological relationships between the nodes). Nodes are displayed using various shapes that represent the functional class of the gene product. The node color indicates up (red)- or down (green)-regulation by Pioglitazone. Edges are displayed with various labels that describe the nature of the relationship between the nodes (A, activation; B, binding; E, expression; I, inhibition; P, phosphorylation; T, transcription). Edges without a label represent binding only. The node *Srebf1* was identified by the pathway analysis as part of the network, and its differential gene expression was determined subsequently by qRT-PCR. A total of 15 differentially expressed focus genes were brought into this network with a highly significant score of 30.

**Figure 3:**
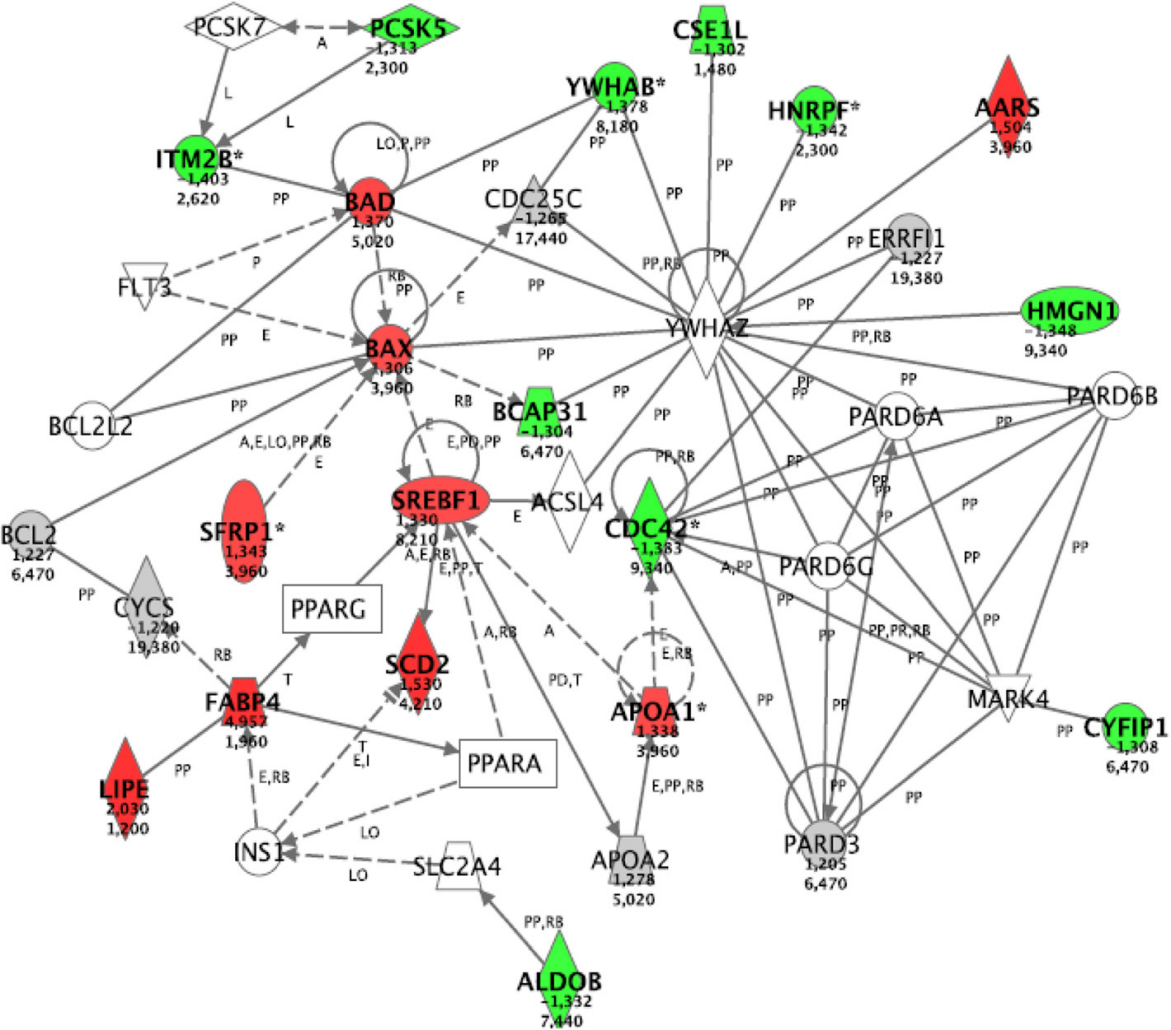
**Network related to lipid metabolism in islets cultured at 23 mM glucose concentration.** For the explanation of the symbols and letters, see the legend to Figure 2. A total of 19 differentially expressed focus genes were brought into this network with a highly significantly score of 22. The node *Srebf1* was identified by the pathway analysis as part of the network, and its differential gene expression was determined subsequently by qRT-PCR. The node *Ywhaz* was tested for being included in the pathway, but it was not Pioglitazone dependent.

**Figure 4:**
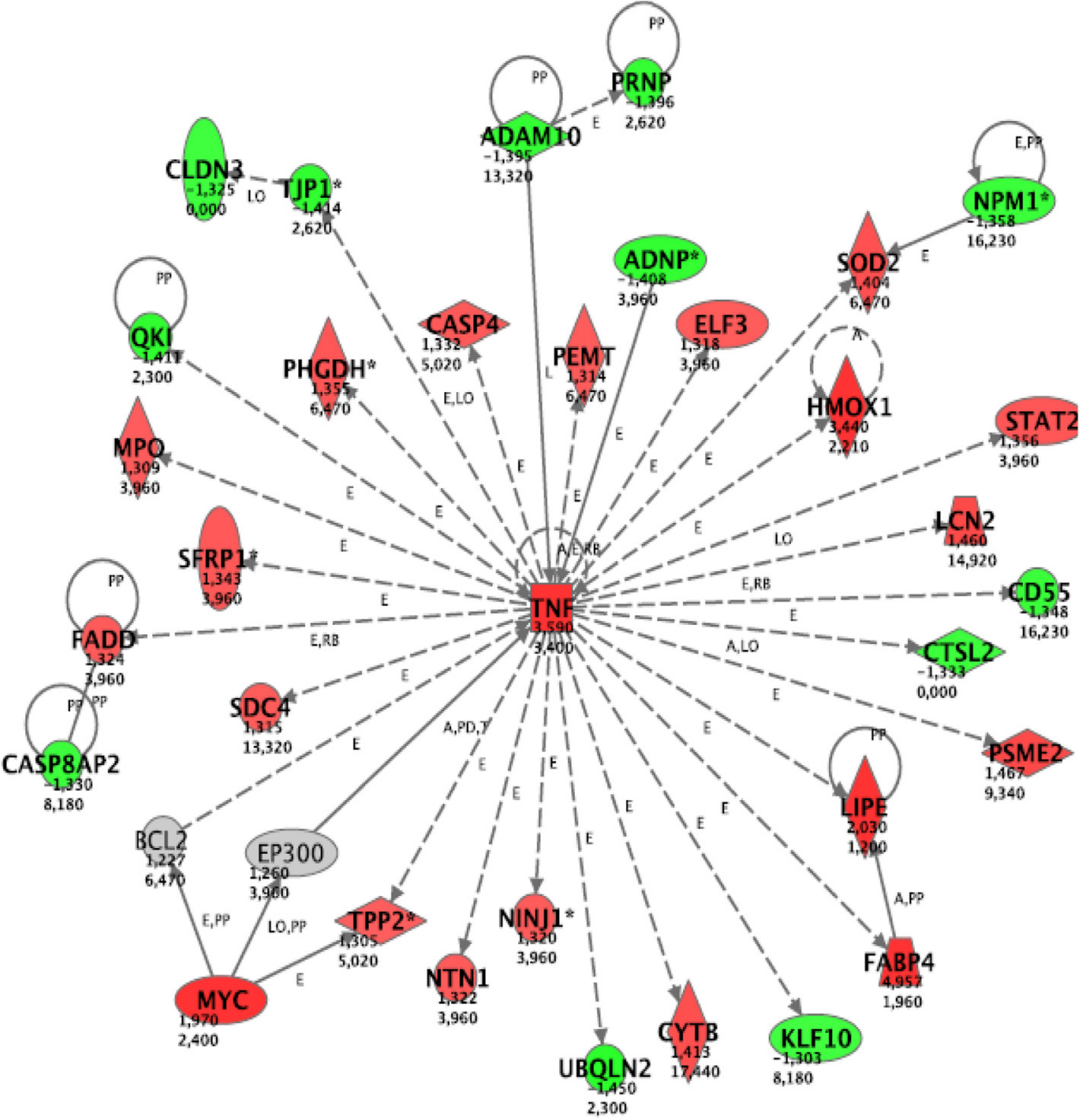
**Network related to cell cycle and cell death in islets cultured at 23 mM glucose concentration.** For the explanation of the symbols and letters, see the legend to Figure 2. A total of 33 differentially expressed focus genes were brought into this network with a highly significant score of 60. The three nodes *Tnf*, *Myc* and *Lipe* were identified by the pathway analysis as part of the network, and their differential gene expression was determined subsequently by qRT-PCR.

Pathway analysis indicated a single gene network, related to lipid metabolism, as Pioglitazone dependent at 5.6 mM glucose (Figure 2). Among the differentially expressed genes included in this network six were confirmed through qRT-PCR. All genes showed the same degree and direction of change, but for *Scd1* and *Sc4mol* qRT-PCR data were not statistically significant (Table 1). At 23 mM glucose the drug effect was much more pronounced, and consequently the number of regulated genes was dramatically increased. The two most strikingly affected networks included genes related to lipid metabolism, and cell death and cell cycle (Figures 3 and 4, respectively). Among the 10 genes selected for validation by qRT-PCR, six genes were confirmed as differentially expressed after Pioglitazone treatment (Table 1). Based upon the networks generated by our pathway analysis, we tested additional key genes by qRT-PCR. As shown in Table 2, all these genes were also Pioglitazone dependent.

**Table 2:** Analysis (by qRT-PCR) of the expression of additional genes identified as Pioglitazone targets by network analysis after 24-hour exposure to the drug at 5.6 mM and 23 mM glucose concentrations

	**Genes**	**FC**	***p*-value**
5,6 mM	*Srebf1*	1.3	0.02
23 mM	*Aldob*	−1.8	0.05
	*Lipe*	2.0	0.03
	*Myc*	2.0	0.04
	*Scd2*	1.5	0.01
	*Srebf1*	1.3	0.03
	*Tnf*	3.6	0.05

Positive and negative FC (fold-change) represent, respectively, genes upregulated and downregulated by Pioglitazone.

### Evaluation of apoptosis

The measurement of insulin secretion performed to rule out a toxic effect of Pioglitazone demonstrated that glucose-stimulated insulin secretion was not significantly different between islets exposed and not exposed to Pioglitazone for 24 h, with stimulation indices of 2.0 ± 0.3 and 1.9 ± 0.4, respectively, in islets maintained at 5.6 mM glucose and of 3.2 ± 1.2 and 2.1 ± 0.9, respectively, in islets maintained at 23 mM glucose.

**Figure 5:**
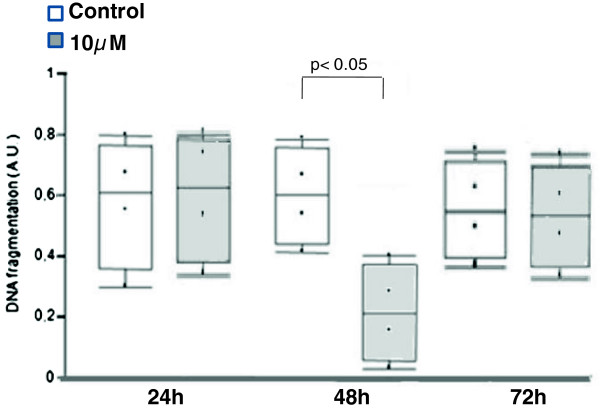
**Apoptosis rate as measured by DNA fragmentation of islets exposed to Pioglitazone at physiological glucose concentration.** Box diagram comparing islet treated by Pioglitazone (10 μM) with non-treated cells (Control). The horizontal line within the box plot represents the median value, the box plot limits refer to 25^th^ to 75^th^ percentiles, and the box plot bars include the 10th to 90th percentiles for apoptosis rate.

**Figure 6:**
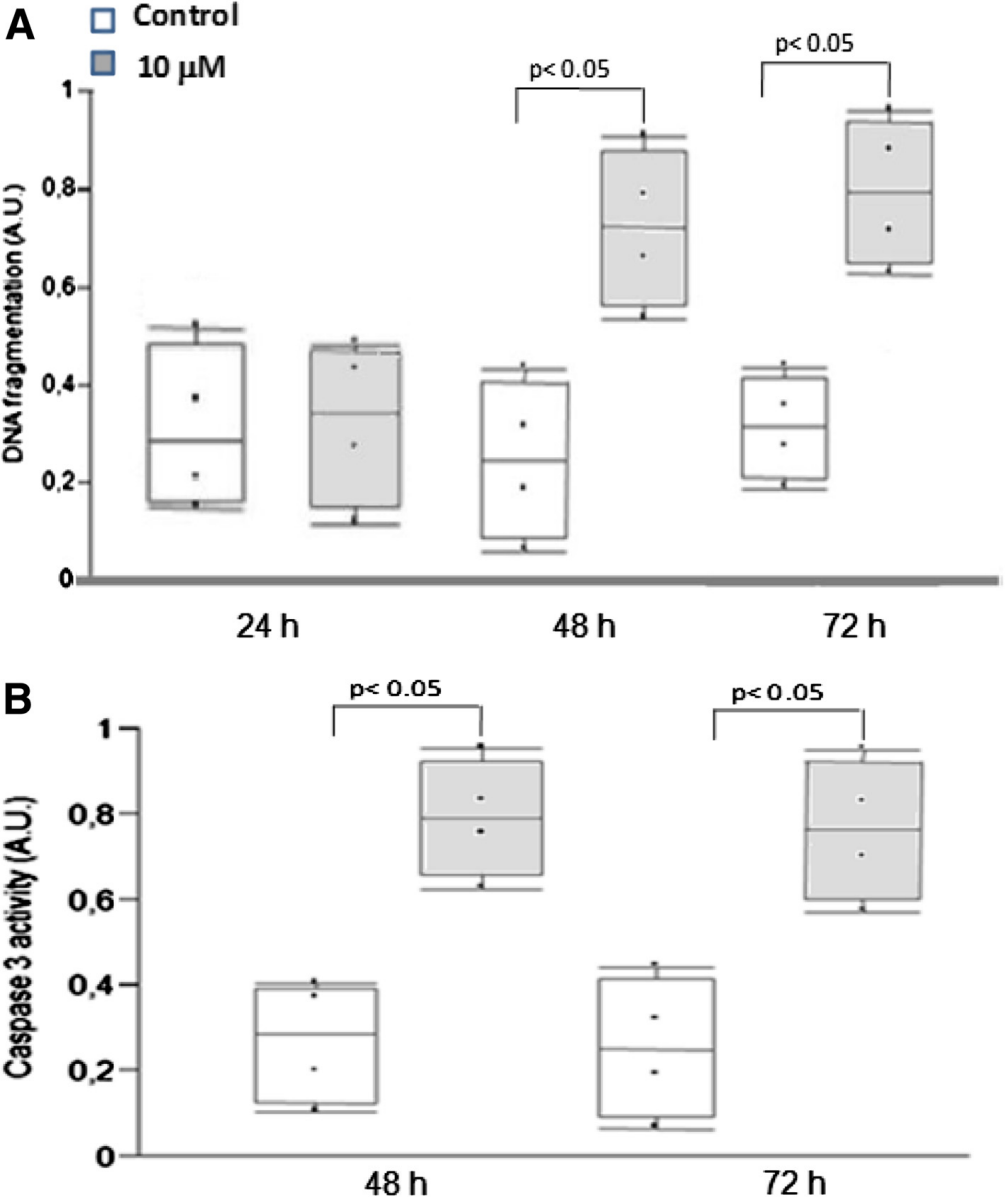
**Apoptosis rate as measured by DNA fragmentation (Panel A) and by caspase 3 activity (Panel B) of islets exposed to Pioglitazone at supraphysiological glucose concentration.** Box diagram comparing islet treated by Pioglitazone (10 μM) with non-treated cells (Control). The horizontal line within the box plot represents the median value, the box plot limits refer to 25th to 75th percentiles, and the box plot bars include the 10th to 90th percentiles for apoptosis rate.

In islets cultured at 5.6 mM glucose, *Bcl2* RNA expression was not significantly modulated by Pioglitazone at any of the evaluated time-points (Table 3). The apoptosis rate was transiently decreased in comparison to untreated islets after 48 h exposure to the drug (Figure 5). At 23 mM glucose, *Bcl2* RNA expression was significantly lower in islets treated with Pioglitazone for 24 and 48 h (Table 3). In agreement with the reduction of expression of this antiapoptotic gene, the apoptosis rate evaluated by DNA fragmentation (Figure 6A) and caspase-3 activity (Figure 6B) was shown to be increased by Pioglitazone after 48 and 72 h.

**Table 3:** **Analysis (by qRT-PCR) of the *Bcl2 *expression induced by 24-, 48- and 72- hour exposure to Pioglitazone at 5.6 mM and 23 mM glucose concentrations**

**Glucose concentration**	**Time(h)**	**FC**	***p*-value**
5.6 mM	24	−1.2	ns
	48	−1.1	ns
	72	1.0	ns
23 mM	24	−3.0	0.001
	48	−2.5	0.002
	72	−1.1	ns

Negative FC (fold-change) represents downregulation by Pioglitazone.

## Discussion

This study, designed to further investigate the direct effects of Pioglitazone on pancreatic islets, demonstrates that the effect of this TZD on islet apoptosis rate and gene expression profile depends on the glucose concentration.

Parton et al. [28] had investigated gene expression profiles of rat islets treated with a PPAR-γ agonist (GW-347845) only at 3 mM glucose and found that 49 out of 9,563 genes exhibited altered expression levels. A substantial increase in the number of genes affected was observed only when islets overexpressing PPAR-γ were treated with GW-347845. Our current finding, in which 101 out of 13,059 genes were affected by Pioglitazone in islets maintained at 5.6 mM glucose, suggests that gene expression in β-cells is not very sensitive to TZDs in euglycemic conditions and in cells with physiological expression of PPAR-γ. A further similarity between both studies was the lack of modulation of the genes coding for glucokinase and UCP2, previously suggested as PPAR-γ targets [29,30].

At both glucose concentrations Pioglitazone increased the expression of *Scd2* and *Fabp4,* lipogenic genes which are PPAR-γ targets in adipocytes [31,32] and *Srebf1*, a transcription factor involved in lipogenesis in adipocytes [33]. In the opposite metabolic direction, Pioglitazone increased the expression of *Insig1* (at 5.6 mM), a protein that participates in the inhibition of cholesterol synthesis [34] and of *Lipe* (at 23 mM), which codes for hormone-sensitive lipase, responsible for hydrolysis of triglycerides [35]. Thus, in conditions of physiological expression of PPAR-γ, Pioglitazone stimulates expression of genes that limit lipid accumulation in islets, but we cannot rule out that these changes are a response to a primary activation of lipogenic pathways, which is more concordant with the role of PPAR-γ in other tissues.

The transcriptional response to Pioglitazone was strikingly different under conditions of elevated glucose, an effect not previously described. Concomitant treatment of islets with Pioglitazone and 23 mM glucose increased by 12-fold the number of modulated genes. Little is known about the mechanisms involved in glucose regulation of β-cell gene expression. It has been suggested that glucose metabolism alters gene expression directly at the transcriptional level by interfering with proteins bound to the preinitiation complex or by modifying the concentrations of transcription factors [36]. Although further studies are necessary to determine the contribution of enhanced RNA stability or direct transcriptional activation on the observed response, it can be hypothesized that at high concentrations, glucose interferes with ligand-dependent PPAR-γ transcriptional activity, directly modulating the expression of genes that are not affected when islets are exposed solely to the PPAR-γ ligand. However, since the transcriptional response to Pioglitazone was determined after 24 h in the absence of protein synthesis inhibitors, it is likely that part of the observed gene regulation is not directly due to ligand binding to PPAR-γ, but resulted from changes in expression of other transcriptional regulators targeted by the combination of glucose and ligand. It is also worth mention that both high glucose concentrations [37] and PPAR ligands [38] promote chromatin remodeling and transcription, which could also contribute to the observed finding.

At 48 h, Pioglitazone elicited a reduction in islets apoptosis rate at 5.6 mM glucose. The transient nature of this response makes it difficult to state that this TZD exerts a protective effect on pancreatic islets, but one can conclude that in such experimental condition, Pioglitazone is not deleterious, a result in contrast to that obtained at supraphysiological glucose concentration, at which an increased apoptosis rate was found after 48 and 72 h. This pro-apoptotic tendency was also revealed by the gene expression analysis after 24 h, since genes related to cell cycle and cell death were significantly modulated. Because genes related to lipid metabolism were also modulated at 5.6 mM, this single pathway does not seem to be related to the apoptotic fate of islets. Additional data that reinforce a pro-apoptotic trend of islets exposed to Pioglitazone at 23 mM glucose are upregulation of antioxidant defense genes such as *Hmox1* and *Sod2*, believed to be activated, respectively, in response to cellular stress [39] and in ß-cell defense [40], and a lower *Bcl2* RNA expression after 48 and 72 h of exposure. Although several studies have already demonstrated that TZD´s may counteract direct fatty acid induced deleterious effects on β-cell function [11,41-44], few studies addressed the direct effects of TZD´s in conditions mimicking hyperglycemia. Zeender et al. had found that Pioglitazone decreased the apoptosis rate of human islets exposed to 33.3 mM glucose after 48 h (5 μM) and 96 h (1 μM) while Saitoh et al. did not observe any effect of Pioglitazone on the apoptotic rate of MIN-6 cells exposed to 25 mM glucose [11] and Ohtani et al. found that troglitazone (10^-4^ to 10^-6^ M) induced apoptosis on HIT-15 cell exposed to 7 mM glucose for 72 h [45]. Reasons for the discrepancies between the studies might be the use of different experimental conditions such as glitazones and glucose concentrations, different cell lines or islets from different species, but the controversial results point out the need for further research on this issue.

It is probable that *in vivo* the indirect beneficial effects of TZDs on islets compensate for the potential deleterious direct effects in the presence of high glucose, given that previous studies have already demonstrated restoration of islet function in rodents [7,8] and humans [4-6]. Furthermore, besides hyperglycemia, other conditions not addressed in this study participate in the loss of β-cell observed in type 2 diabetes, and there are reports supporting a protective effect of TZDs in presence of lipotoxicity [44,46,47], deposition of islet amyloid [48] and endoplasmic reticulum stress [38]. In agreement with the present study, however, a former *in vitro* study has already found that troglitazone protective effects on islets were less pronounced at high glucose [49].

In summary, our data demonstrate for the first time that the effect of Pioglitazone on pancreatic islet gene expression profile and apoptosis rate depends on the glucose concentration, reinforcing the necessity of additional studies designed to evaluate TZDs effects on the preservation of β-cell function in situations where glucotoxicity might be more relevant than lipotoxicity.

## Abbreviations

TZD: Thiazolidinedione

## Competing interests

No potential conflict of interest relevant to this article is reported.

## Authors’ contributions

RNL performed microarray and RT-PCR experiments, CNC performed apoptosis and insulin secretion experiments, PW provided input into the design of the study and performed microarray statistical analysis; FLC and LSO helped in islet isolation; DGN provided input into the design of the study and performed statistical analysis; KHK participated in the design, helped funding the study and revised the manuscript; MLCG participated in the design of the study, obtained funding and wrote the manuscript. All authors read and approved the final manuscript.

## Supplementary Material

Additional file 1: Table S1 Primers used in the qRT-PCR assays. Click here for file

Additional file 2: Table S2 List of genes which were down- and up-regulated by Pioglitazone in islets maintained at physiological glucose concentration. Click here for file

Additional file 3: Table S3 List of the first 200 genes which were down- and up-regulated by Pioglitazone in islets maintained at supra-physiological glucose concentration. Click here for file
