# Supplementary figures and images for: Apoptosis rate and transcriptional response of pancreatic islets exposed to the PPAR gamma agonist Pioglitazone
Source: Diabetol Metab Syndr. 2013 Jan 8;5:1. doi: 10.1186/1758-5996-5-1 (PMC3598339; doi:10.1186/1758-5996-5-1)

**Table supplemental 1: Primers used in the qRT-PCR assays**


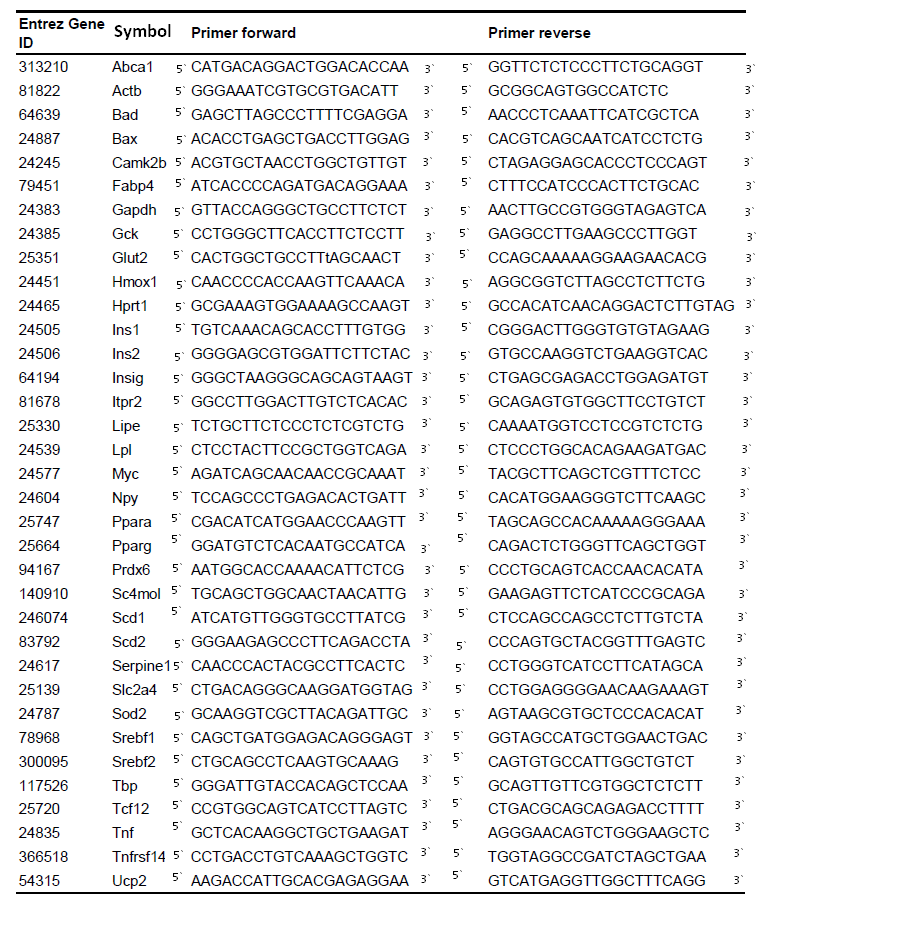

Supplement: Additional file 1: Table S1 — Primers used in the qRT-PCR assays. [file 1758-5996-5-1-S1.doc]
