# Supplementary material for: Apoptosis rate and transcriptional response of pancreatic islets exposed to the PPAR gamma agonist Pioglitazone
Source: Diabetol Metab Syndr. 2013 Jan 8;5:1. doi: 10.1186/1758-5996-5-1 (PMC3598339; doi:10.1186/1758-5996-5-1)
Supplement: Additional file 3: Table S3 — List of the first 200 genes which were down- and up-regulated by Pioglitazone in islets maintained at supra-physiological glucose concentration. [file 1758-5996-5-1-S3.doc]

**Table supplemental 3: List of the first 200 genes which were down- and up-regulated by Pioglitazone in islets maintained at supra-physiological glucose concentration (23 mM).**

**
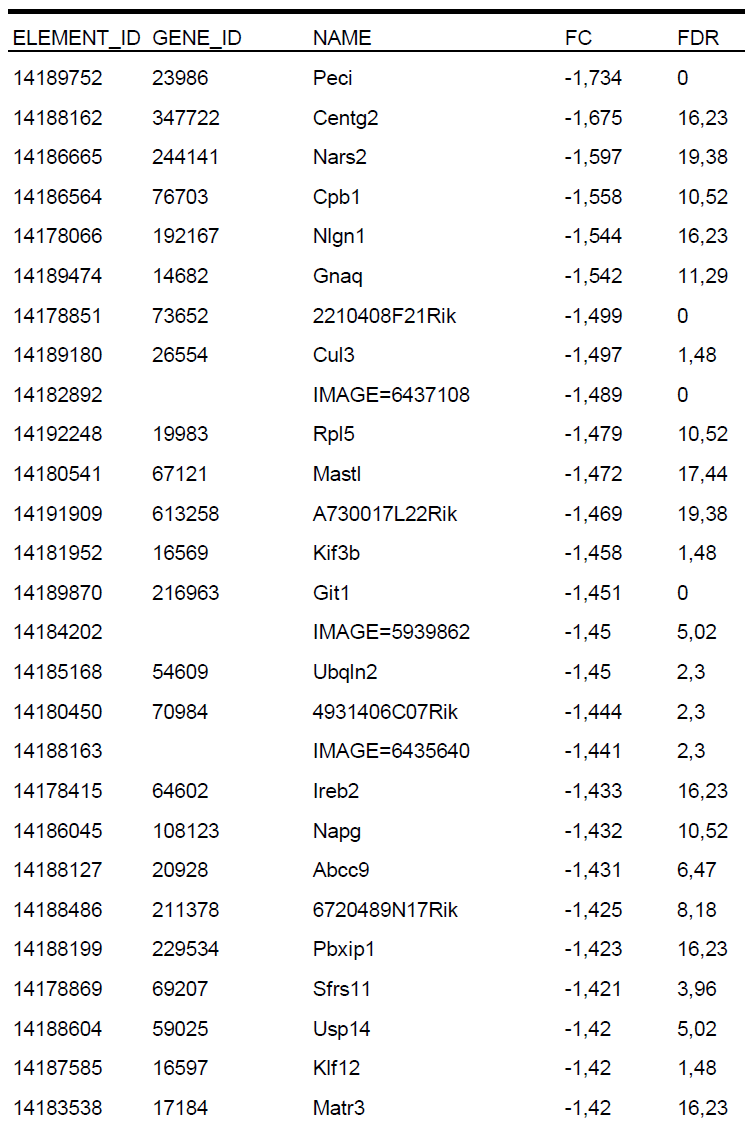
**


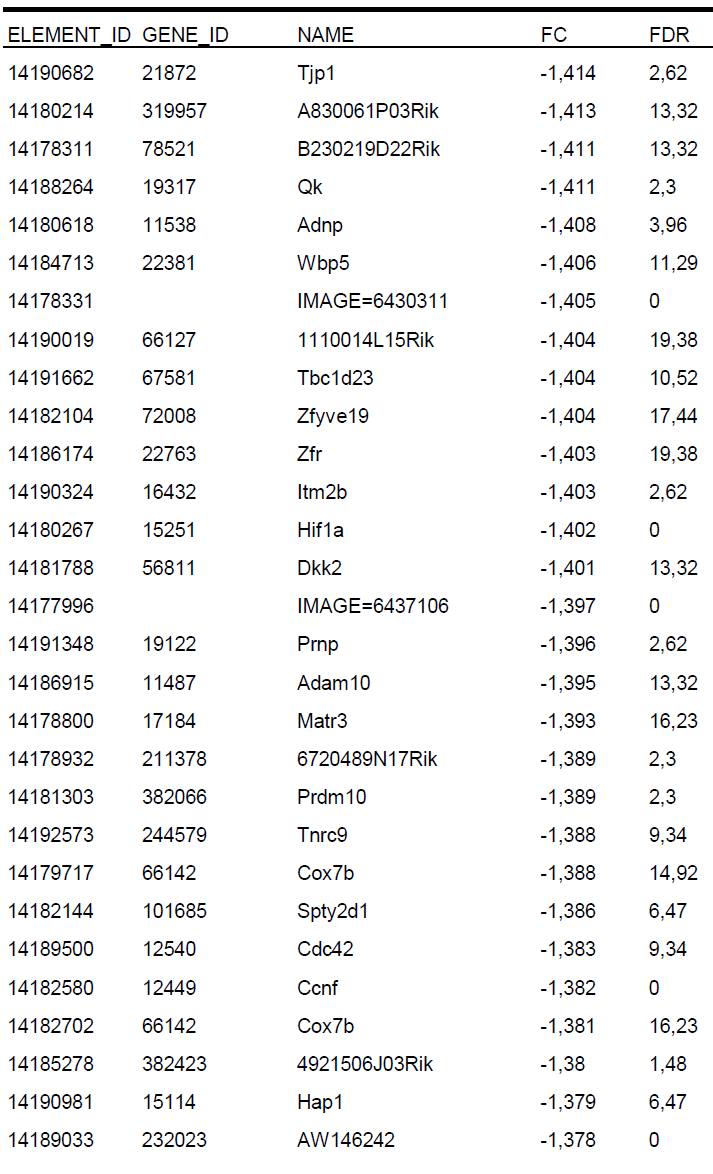


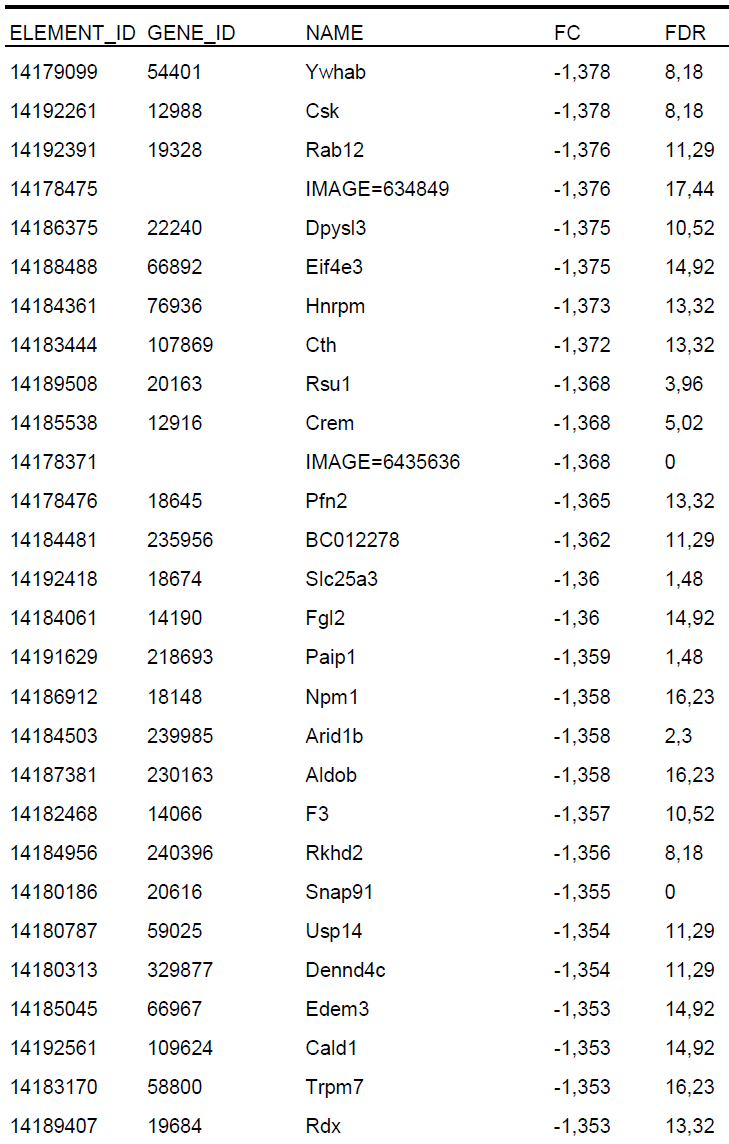


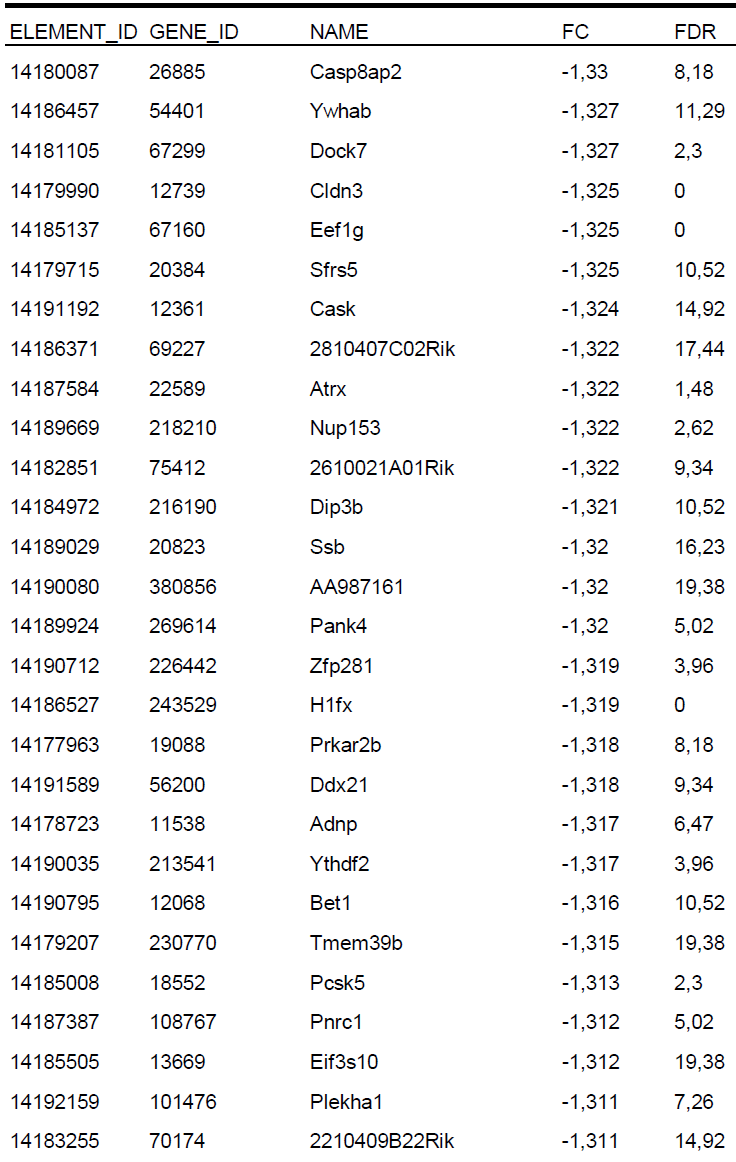


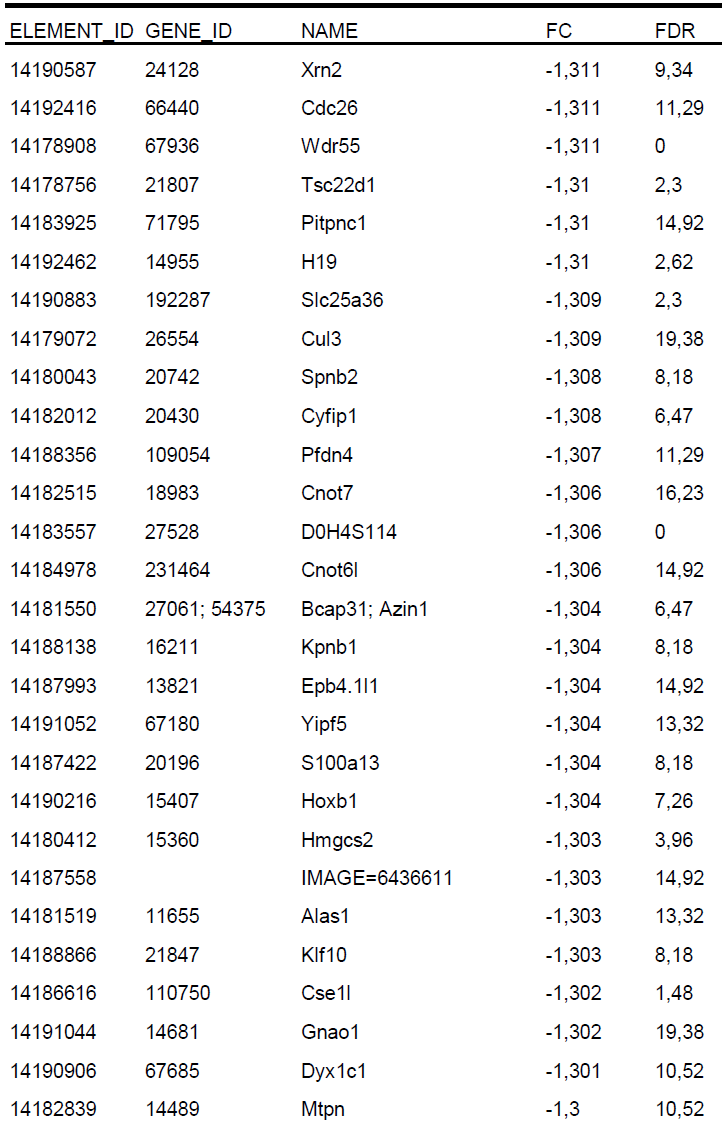


Positive and negative FC (fold-change) represent, respectively, genes upregulated and downregulated by Pioglitazone. FDR: false-discovery rate.
